# Polyunsaturated Fatty Acids (PUFAs): Sources, Digestion, Absorption, Application and Their Potential Adjunctive Effects on Visual Fatigue

**DOI:** 10.3390/nu15112633

**Published:** 2023-06-05

**Authors:** Hao Duan, Wei Song, Jian Zhao, Wenjie Yan

**Affiliations:** 1 College of Biochemical Engineering, Beijing Union University, Beijing 100023, China; dhuanao@163.com; 2 Beijing Key Laboratory of Bioactive Substances and Functional Food, Beijing Union University, Beijing 100023, China

**Keywords:** visual fatigue, PUFAs, retina, ocular surface, safety, absorption, mechanism

## Abstract

When the eyes are exposed to the environment, they are easily affected by strong light stimulation and harmful substances. At the same time, prolonged use of the eyes or incorrect eye habits can cause visual fatigue, which mainly manifests as eye dryness, soreness, blurred vision, and various discomforts. The main reason for this is a decline in the function of the eye, especially the cornea and retina on the surface of the eye, which have the greatest impact on the normal function of the eye. Research has found that supplementation with appropriate foods or nutrients can effectively strengthen the eye against external and internal stimuli, thereby alleviating or avoiding visual fatigue. Among these, supplementation with polyunsaturated fatty acids has been found to be effective at protecting eye health and relieving visual fatigue. This article summarizes the sources of polyunsaturated fatty acids (including the main dietary sources and internal synthesis), the mechanisms of digestion and absorption of polyunsaturated fatty acids in the body and the safety of polyunsaturated fatty acid applications. It also reviews the mechanism of action of polyunsaturated fatty acids in aiding the relief of visual fatigue based on the mechanism of impaired function or structure of the ocular surface and fundus in the hope of providing some reference and insight into the development and application of polyunsaturated fatty acids in functional foods for the relief of visual fatigue.

## 1. Introduction

The eye is a very delicate and complex biological organ with precise and integrated light-sensitive and visual processing functions. At the same time, the eye is richly vascularized and neurologically distributed, resulting in a higher demand for oxygen and the consequent production of metabolic products [1], as well as a greater susceptibility to oxygen free radical attacks. In addition, other factors, such as lighting, nature of the work involved, amount of time, distance, and habits, can further exacerbate the burden on the eye, resulting in various forms of discomfort and symptoms of visual fatigue, such as blurred vision, that affect people’s normal visual function [2]. Visual fatigue, also known as eyestrain, is common among people who frequently use electronic products or have incorrect eye habits [3]. Currently, the number of people with visual fatigue is increasing every year [4]. According to the Vision Council, at least 60% of American men and 65% of American women experience visual fatigue, 80% of adults use digital devices for at least 2 h a day, and over 65% of adults use at least two devices at the same time. Approximately 80% of adults use digital devices at bedtime, and more than 70% of adults indicate that their children’s electronic screen exposure is well above 2 h [5,6]. Moreover, numerous studies have shown that using multiple electronic devices at the same time for work or study often increases the incidence of visual fatigue [4,7]. One study reported that the incidence of visual fatigue among university students around the world was approximately 46–71% [8]. People who work or study with electronic devices for long periods of time and older people whose eye function has declined because of aging are vulnerable to visual fatigue [3,9,10], which can also affect their physical and mental health in severe cases.

The main cause of visual fatigue is structural damage or reduced function of the ocular surface and fundus, especially damage to the retinal function of the fundus, which predisposes to the development of visual fatigue [11,12]. Vision begins in the retina, which is neuronal tissue that perceives and receives external light, processes it into electrical signals, and transmits it to the brain to obtain images [13,14]. When the retinal pigment epithelium (RPE) cell structure and function is damaged, this will seriously affect retinal function [15]. PRE cells are very active, consume high amounts of energy and nutrients, and demand a large amount of oxygen, so they are prone to producing reactive oxygen species (ROS), a process that can lead to oxidative stress if ROS are not processed in time. Especially, when the eye is exposed to prolonged bright light, intensive light or low illumination, the burden on the retina is more likely to increase, and damage to the eye due to oxidative stress becomes more severe [16] and visual fatigue ensues. Studies have shown that the retina contains high concentrations of docosahexaenoic acid (DHA), which is important for the survival of photoreceptor cells. In the disc membrane of photoreceptors, DHA can account for up to 50% of the total fatty acid content of phospholipids [17], and it is also an important substance involved in the phototransduction cascade reaction [18], which shows the necessity and importance of DHA for eye health. On the other hand, however, polyunsaturated fatty acids (PUFAs) such as DHA are susceptible to oxidation by peroxides and produce large amounts of lipid peroxides, which can subsequently cause a series of inflammatory injuries and apoptotic responses in cells [19], leading to a further increase in visual fatigue [20]. In addition, aging of the RPE also contributes to a decrease in the macular pigment optical density (MPOD), which affects normal visual function and also makes it more likely that visual fatigue will develop [21]. Adequate omega-3 supplementation for the eye has been reported to reduce this range of adverse reactions, thereby alleviating the degree of visual fatigue [22]. Secondly, the cornea on the ocular surface is an important refractive medium and is key to protecting the homeostasis of the ocular surface [23]; however, the ocular surface is exposed to the external environment for long periods of time and is susceptible to a series of adverse environmental effects, such as bright external light, high concentrations of oxygen and excessive dryness, and further induces structural and functional damage to the ocular surface, resulting in reduced tear volume and tear quality, frequent dry eyes, ocular discomfort [24,25], and visual fatigue The result is reduced tear volume, reduced tear quality, frequent dry eyes and eye discomfort [24,25], and visual fatigue. In conclusion, damage to the structure or function of the fundus and ocular surface, as well as aging of the eye, are important causes of visual fatigue.

Therefore, effective nutritional intervention strategies should be proposed for improving or reducing the degree of damage to the fundus or ocular surface and balancing the health of the internal environment of the eye, thus playing an adjunctive role in relieving visual fatigue. In recent years, an increasing number of studies have found that optimal visual performance can be effectively promoted by supplementing the diet with appropriate foods or nutrients [22], such as anthocyanins [26], taurine [27], DHA- and eicosapentaenoic acid (EPA)-rich fish oils, lutein [28], astaxanthin [29], and lingonberry extracts [30], which can reduce or alleviate the onset of visual fatigue [31]. Among them, PUFAs, as essential nutrients, have a very important supporting role in alleviating visual fatigue [32]. Therefore, this paper summarizes the sources of PUFAs (including the main dietary sources and internal synthesis), the mechanisms of digestion and absorption of PUFAs in the body, and the safety of PUFA application. It also reviews the mechanism of action of polyunsaturated fatty acids in aiding the relief of visual fatigue based on the mechanism of impaired function or structure of the ocular surface and fundus in the hope of providing a basis of reference and insight into the development and application of polyunsaturated fatty acids in functional foods for the relief of visual fatigue.

## 2. Sources of PUFAs

**Figure 1:**
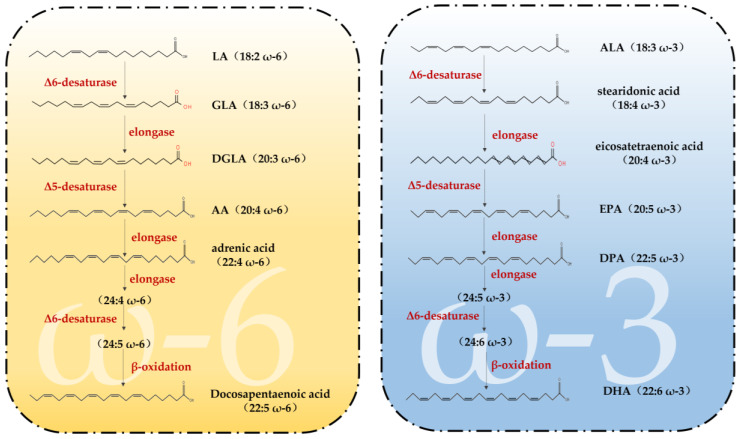
Synthetic pathways of ω-3 PUFAs and ω-6 PUFAs (adapted from [23]). LA = linoleic acid; GLA = gamma-linolenic acid; DGLA = dihomo-γ-linolenic acid; AA = arachidonic acid; ALA = Alpha-linolenic acid; EPA = eicosapentaenoic acid; DPA = docosapentaenoic acid; DHA = docosahexaenoic acid.

**Figure 2:**
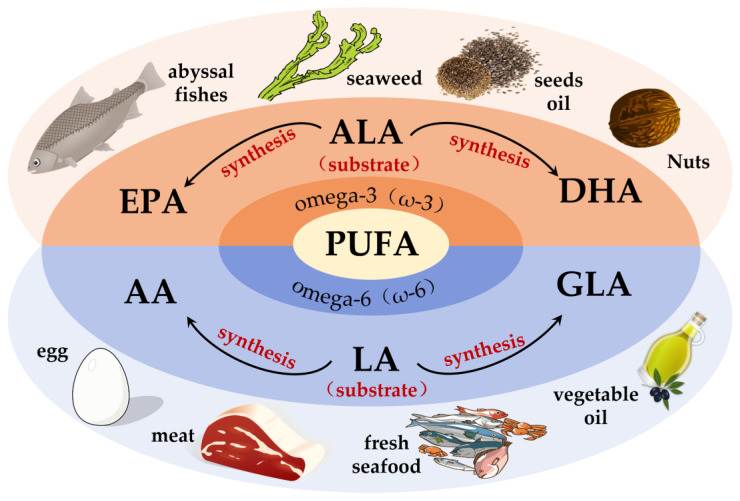
Major membership and main dietary sources of ω-3 PUFAs and ω-6 PUFAs. DHA = docosahexaenoic acid; ALA = alpha-linolenic acid; EPA = eicosapentaenoic acid; GLA = gamma-linolenic acid; LA = linoleic acid; AA = arachidonic acid.

Fatty acids (FAs) can be classified into three categories based on the number of double bonds present in their side chains: saturated fatty acids, monounsaturated fatty acids and polyunsaturated fatty acids [33]. Among them, PUFAs are molecules that contain two or more double bonds [34]. In addition, the higher the number of double bonds, the more likely they are to produce lipid peroxidation. Data show that two families of PUFAs, OMEGA-3 (ω-3) and OMEGA-6 (ω-6), have important effects on the physiological health of the body [35], including ALA in ω-3 PUFAs, which can be used as a synthetic substrate for ω-3 PUFAs, such as EPA and DHA in organisms; LA in ω-6 PUFAs, which can be used for the synthesis of ω-6 PUFAs, such as arachidonic acid (AA) and gamma-linolenic acid (GLA) [34]. Figure 1 shows the pathways of ω-3 and ω-6 synthesis via substrates. As these two families of substrates do not synthesize sufficient amounts of other PUFAs to support all life activities in the body, linoleic acid (LA) and alpha-linolenic acid (ALA) are also known as essential fatty acids, i.e., the body also needs to supplement them from the dietary pathway [36]. It has been reported that the body can only convert very small amounts of fatty acids into PUFAs with more than 20 carbon atoms, and humans must consume foods containing more than 20 carbon atoms of PUFAs through the diet to meet the normal physiological needs of the body. This also suggests the importance of rational supplementation of foods rich in PUFAs for the maintenance of health and life activities. The main members of ω-3 and ω-6 and the main dietary sources are detailed in Figure 2. The contents of ω-3 PUFAs and ω-6 PUFAs in selected plant and animal foods are shown in Table 1 [33].

## 3. Mechanisms of Digestion and Absorption of PUFAs in the Organism

PUFAs are long-chain fatty acids and their digestion process is more complex. First, lipid food bases containing PUFAs enter the mouth and are initially broken down under conditions of mastication and the involvement of lingual lipases [54,55]. Next, lipid-based food bases containing PUFAs are propelled through the esophagus to the stomach and further hydrolyzed by gastric lipases in gastric juice [56]. Subsequently, the pre-digested lipids from the stomach are pushed into the duodenum where they are hydrolyzed and digested by pancreatic lipase [57]. Ultimately, the digestion products of lipids, such as PUFAs, including monoglycerides, cholesterol, free fatty acids, and other substances, are emulsified by bile salts and phospholipids to form mixed micelles, thus facilitating absorption and transport [58,59,60]. Throughout the digestion process, the digestibility of lipids decreases with an increasing fatty acid chain length, in descending order of digestibility: short-chain fatty acids, medium-chain fatty acids and long-chain fatty acids [61]. In addition, factors such as co-food base composition and organismal differences can affect the rate of this process [62].

**Figure 3:**
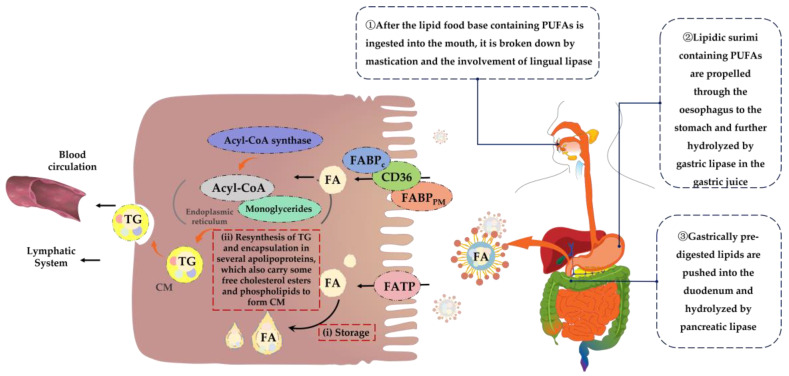
Mechanism of digestion and absorption of dietary PUFAs. FA = fatty acid; TG = triglyceride; CM = chylomicron.

The mechanisms of fatty acid absorption are not yet fully understood. However, it is possible to identify two links between the mode of absorption of mixed micelles at the brush border of the small intestinal epithelium and their release from the basolateral membrane as chyme particles, which are key to fatty acid absorption [63]. Because of their hydrophobic nature, fatty acids are usually bound to serum albumin and cytoplasmic fatty acid-binding protein (FABP) [64]. Therefore, passive diffusion may be the main route of fatty acid uptake [65]. However, there is increasing evidence to support that long-chain fatty acids such as PUFAs also require proteins to facilitate their entry into the small intestinal epithelium [66,67], mainly fatty acid translocase (FAT/CD36), plasma membrane-associated fatty acid-binding protein (FABP_PM_) and the fatty acid transport proteins (FATP) family [68,69]. Fatty acids entering the enterocyte can be temporarily stored in cytoplasmic lipid droplets or transferred to the endoplasmic reticulum via the cytoplasmic fatty acid-binding protein FABP_C_, which is activated by acyl-coenzyme A (Acyl-CoA) synthase, forming fatty Acyl-CoA and binding to monoglycerides to resynthesize triglycerides [70,71] and encapsulated in several apolipoproteins that also carry some of the free cholesterol esters and phospholipids, forming celiac particles [72], which are transported into the lymphatic system and eventually released into the blood. During this process, surrounding cells can take up the required fat from celiac particles [73,74]. Figure 3 shows the mechanism of digestion and absorption of dietary PUFAs in the organism.

## 4. Application Safety of PUFAs

Both ω-3 PUFAs and ω-6 PUFAs have a wide range of physiological functions, but the functions are distinctly different and often show opposite effects. For example, diets rich in ω-6 PUFAs are positively associated with inflammation and platelet aggregation [75], and a normal inflammatory response protects the body from infection and injury [75], but excessive and persistent inflammatory stimulation is greatly associated with cancer risk [76]. In contrast, ω-3 PUFAs have been negatively associated with inflammation, cardiovascular disease and cancer [77,78]. However, the key to the beneficial or harmful effects of both is the amount consumed, which suggests that studies on the safety of consumption of both are necessary. However, there have been no cases of safety incidents with PUFAs, suggesting that the consumption of PUFAs for health management strategies is safe and feasible. Table 2 summarizes the adequate intake (AI) of ω-3 PUFAs and ω-6 PUFAs by food authorities in a number of countries for their population. As can be seen from Table 2, there is some variation in the recommended AI across countries and some differences between men and women. This may be related to ethnicity, environment, and diet structure.

## 5. Study on the Mechanism of PUFAs to Assist in the Relief of Visual Fatigue

**Figure 4:**
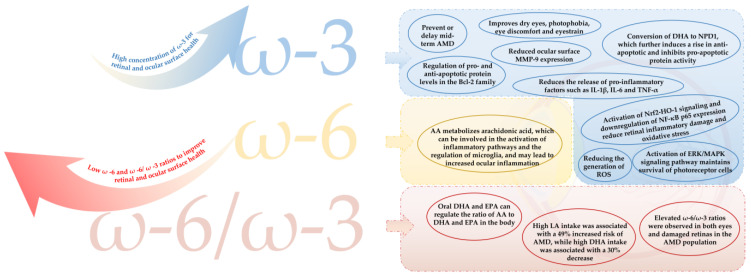
The main mechanism of PUFAs in relieving asthenopia. AMD = age-related macular degeneration; MMP-9 = matrix metalloproteinase-9; NPD1 = neuroprotection D1; IL-1β = interleukin-1β; IL-6 = interleukin-6; TNF-α = tumor necrosis factor-α; AA = arachidonic acid; NF-κB = nuclear factor kappa-B; ROS = reactive oxygen species; DHA = docosahexaenoic acid; EPA = eicosapentaenoic acid; LA = linoleic acid.

The main causes of visual fatigue are related to structural damage or reduced function of the cornea at the surface of the eye and the retina at the base of the eye [24,25,31]. The main mechanisms by which PUFAs relieve visual fatigue are detailed in Figure 4.

### 5.1. PUFAs Improve Damaged Ocular Surfaces to Act as a Visual Fatigue Reliever

The cornea on the ocular surface is rich in sensory nerves and is an important refractive medium and key to maintaining ocular surface homeostasis [23]. However, the ocular surface is highly exposed to the outside world, and strong light and oxygen stimuli in the environment tend to damage the structure and function of the cornea, resulting in reduced tear volume, increased corneal sensitivity [23,24,25,85] and further dry eye, which can lead to the onset of a range of visual fatigue symptoms, such as photophobia and blurred vision. ω-3 PUFAs and ω-6 PUFAs are important components of the cellular phospholipid membrane and are thought to be critical for maintaining cellular function [86]. It has been reported that the intake of ω-3 PUFAs may affect the neural architecture of the cornea and have an important role in corneal health [87]. Dietary supplementation with LA and GLA from ω-6 PUFAs has also been shown to promote ocular surface tear production and reduce dry eye after refractive keratectomy [88,89], thereby alleviating the onset of visual fatigue. Currently, most studies support that high levels of ω-3 PUFAs show a negative correlation with the occurrence of dry eye, while a high a ω-6: ω-3 ratio shows a positive correlation with the frequent occurrence of dry eye and ocular discomfort [90]. Clinical studies have shown that ω-3 PUFAs can reduce ocular surface inflammation in dry eye populations [91,92], thus effectively alleviating visual fatigue. Meanwhile, a double-blind clinical trial found that continuous oral administration of ω-3 PUFAs (360 mg/d EPA + 240 mg/d DHA) for 30 days significantly reduced the tear evaporation rate and increased tear secretion on the ocular surface in dry eye patients, effectively relieving dry eye and ocular discomfort [93]. In addition, oral administration of ω-3 PUFAs significantly improved the reduction of tear osmolarity and TBUT scores in the dry eye population, suggesting that ω-3 PUFAs improved the intrinsic stability of the tear film. Data show that matrix metalloproteinase-9 (MMP-9), a protein hydrolase, on the ocular surface induces inflammatory cytokines and inflammatory responses, playing an important role in dry eye and ocular surface diseases [94]. This trial also demonstrated that oral administration of ω-3 PUFAs significantly reduced MMP-9 and the Ocular Surface Disease Index (OSDI) score. This supports the ability of ω-3 PUFAs to reduce hyperosmotic stress on the ocular surface epithelium while also directly reducing ocular surface inflammation [95], thus effectively reducing visual fatigue and ocular discomfort. Meanwhile, growing evidence confirms that combined supplementation with ω-3 PUFAs and ω-6 PUFAs has a more significant effect on improving ocular surface health and relieving ocular surface dryness than single supplementation with ω-3 PUFAs. An animal study showed that combined supplementation with ω-3 PUFAs and ω-6 PUFAs (EPA + DHA and GLA) was shown to significantly improve elevated intraocular pressure (IOP) in rats compared to single PUFAs supplementation (EPA and DHA or GLA) and further prevented IOP elevation-induced retinal cell structure and glial cell activation [96]. In a double-blind, randomized and controlled trial, 138 people with dry eyes were given capsules of a supplement enriched with ω-3 PUFAs and ω-6 PUFAs, vitamins and zinc that were to be taken three times daily for three months. The results showed that the supplement enriched with ω-3 PUFAs and ω-6 PUFAs significantly reduced the expression of human leucocyte antigen-DR (HLA-DR), an inflammatory marker of conjunctival epithelium, in the dry eye population, with some improvement in dry eye discomfort [97].

In summary, oral supplementation with PUFAs, especially ω-3 PUFAs with DHA and EPA, may have the effect of improving the stability of the tear film and tear volume and reducing the amount of MMP-9 expression on the ocular surface to reduce the degree of inflammation, thus exerting the effect of relieving visual fatigue. In addition, the combined supplementation of ω-3 PUFAs with ω-6 PUFAs showed better improvement of the ocular surface compared to single supplementation with ω-3 PUFAs, suggesting that the rationed intake of ω-3 PUFAs with ω-6 PUFAs is more beneficial in helping eye health.

### 5.2. PUFAs Improve Damaged Eye Fundus to Exert Visual Fatigue Relief

PUFAs are one of the important components in maintaining retinal homeostasis, as well as promoting its healthy development [98]. The retina is rich in several PUFAs, such as DHA and AA, the former of which is important for maintaining the survival of photoreceptor cells [19] via a possible mechanism that promotes the survival of rat retinal photoreceptors in vitro and under oxidative stress conditions through the activation of the ERK/MAPK signaling pathway [99]. Alternatively, it selectively protects photoreceptors from oxidative stress by regulating the levels of pro- and anti-apoptotic proteins of the Bcl-2 family [100]. In addition, DHA can be converted into neuroprotection D1 (NPD1) via 15-lipoxygenase-1 which, in turn, can further induce anti-apoptotic and inhibit pro-apoptotic proteins, thus exerting a cytoprotective, anti-inflammatory and pro-survival repair signal on photoreceptors. Moreover, a significant decrease in DHA and a significant increase in the ω-6/ω-3 ratio could be observed in the eyes of mice with damaged retinas [19]. This reflects the essential and important role of DHA in the retina. Conversely, the AA metabolism of arachidonic acids, including prostaglandins, coagulants, leukotrienes, and lipoxygenase, is associated with th activation of inflammatory pathways and regulation of microglia [101,102,103].

As ω-3 PUFAs synthesized by the body are not sufficient to sustain all vital activities of an organism, the dietary supplementation route of PUFAs is particularly important [104]. Dietary supplementation with ω-3 PUFAs has been reported to be effective in preventing retinal damage, thus further exerting its effect on visual fatigue. Flaxseed oil rich in ω-3 PUFAs has been reported to protect visual function by activating Nrf2-HO-1 signaling and down-regulating NF-κB p65 expression to reduce visible light-induced oxidative stress and inflammatory damage in the retina [105]. Clinical studies have further confirmed that after a 30 d period of flaxseed oil soft gel supplementation administered to 101 visually fatigued individuals, visual fatigue symptoms were significantly reduced, with a 70.3% persistence of binocular vision and a 51% total effective rate, a significant difference, confirming that dietary supplementation with ω-3 PUFAs can effectively alleviate visual fatigue [106]. Fuminori et al. showed [28] that continuous supplementation with ω-3 PUFAs rich in ω-3 PUFAs, particularly a combination preparation of fish oil (DHA 783 mg/d and EPA 162 mg/d), lingonberry extract (containing anthocyanins 59 mg/d) and luteolin (17.5 mg/d), for 4 weeks significantly improved symptoms in people with visual fatigue. Thus, a growing body of research supports that increasing dietary levels of ω-3 PUFAs can promote human health. It has also been confirmed that dietary supplementation with ω-3 PUFAs is important in preventing or reducing ocular inflammation, e.g., supplementation with fish oil rich in DHA and EPA has been shown to reduce retinal damage in premature infants, thereby repairing impaired visual function in infants [107]. Moreover, the dietary supplementation route of fish oil is also effective in reducing the production of ROS and decreasing the release of pro-inflammatory factors, thus effectively suppressing the levels of inflammatory factors in the retinal vascular endothelium [21]. Clinical studies have also shown that a 3-month oral supplementation with omega-3 (EPA and DHA) significantly inhibited the production of interleukin-1β (IL-1β), interleukin-6 (IL-6) and tumor necrosis factor-α (TNF-α) in blood mononuclear cells in young and elderly women [21]. The expression of pro-inflammatory factors, such as interleukin-1β (IL-1β), interleukin-6 (IL-6) and tumor necrosis factor-α (TNF-α) [108]. Furthermore, in animal studies, it was observed that the plasma production of IL-1β, IL-6 and TNF-α was significantly reduced in C57BL/6 mice after 5–6 weeks of dietary supplementation with EPA + DHA or fish oil [109,110].

In addition, aging is accompanied by a decline in RPE cell viability, which leads to a weakening of their antioxidant and self-healing capacity, making the retinal macula more susceptible to adverse effects and resulting in age-related macular degeneration (AMD), which further exacerbates visual fatigue [24,111]. Epidemiological, clinical and experimental studies have shown that supplementation with omega-3 PUFAs is negatively associated with AMD, while an increase in omega-6 PUFAs increases this risk [112]. Higher levels of EPA and DHA intake were both shown to prevent or delay the onset of AMD in over the medium term [113].

The combined intake of ω-6 and ω-3 has been reported to significantly modulate the amount or ratio of PUFAs in the body, thus reducing the extent of damage to the retina [114,115]. Dietary supplementation with DHA and EPA can effectively regulate the ratio of AA, DHA and EPA in the body [116]. High levels of LA intake can significantly reduce the risk of AMD; conversely, high levels of DHA intake are negatively associated with the risk of AMD [117]. In addition, EPA + DHA + GLA diet significantly reduced the activation of glial cells in retina-damaged rats compared to EPA + DHA or GLA supplementation alone, and it was effective at protecting the structural integrity of the retina [96]. These studies suggest that diets with different combinations of ω-3 and ω-6 PUFAs may be more helpful at improving damaged retinas than single ω-3 PUFAs or ω-6 PUFAs supplementation, suggesting that the modulation of ω-3 PUFAs levels and the ratio of ω-6/ω-3 PUFAs are important in the function of retinal protection. A stable ω-6/ω-3 ratio in the range of 1:1 to 4:1 in the diets of animals and humans has been reported to be an appropriate balance ratio and more conducive to the health of the organism [118]. However, an optimal range for the ω-6/ω-3 ratio has not been clearly reported in studies on the eye.

In summary, oral supplementation with ω-3 PUFAs, especially DHA and EPA, is effective not only in relieving visual fatigue, but also in ameliorating dry eyes [119] or other adverse effects that occur in the eye [120]. These studies also suggest, once again, that the dietary supplementation of ω-3 PUFAs can exert effects on visual fatigue through multiple pathways, including antioxidant and anti-inflammatory, as well as anti-apoptotic, and that the ratio of the ω-3 PUFAs to ω-6 PUFAs intake has important implications for eye health and the extent of visual fatigue-relieving effects, but there appear to be no reported studies examining the optimal ratio of the two in eye health. This may be a direction for future research. Table 3 summarizes the mechanisms by which PUFAs improve the extent of damage to the ocular surface and fundus, thus exerting a visual fatigue-relieving effect.

## 6. Conclusions

As important nutritional components in the retina, PUFAs are integral to ensuring the survival of photoreceptors and participate in visual transmission. Existing research shows that ω-3 PUFAs have good health promoting effects on the retina, especially DHA and EPA, which have been proven to repair or improve damaged retinal conditions through dietary supplementation by activating pathways such as antioxidant, anti-inflammatory or anti-apoptotic. However, currently, there are many gaps in the basic research on the alleviation of visual fatigue with PUFAs. For example, the absorption, transport and metabolic patterns of PUFAs in the body have not been fully revealed; the mechanism and complementary research on the function of PUFA raw material of in relieving visual fatigue is not sufficiently in depth; and research on the physiological mechanisms underlying visual fatigue is not deep enough, which limits the research on the mechanism of PUFA raw materials in relieving visual fatigue, and also overlooks the application potential of PUFA raw materials in relieving visual fatigue.

Therefore, in order to fully utilize PUFA raw materials in the development of visual fatigue relief health food in the future, research on PUFA raw materials should be intensified in four aspects: (1) fully studying the absorption, transport and metabolic mechanism of PUFA raw materials in the body; (2) conducting in-depth research on the mechanism of PUFA raw materials to alleviate visual fatigue; (3) strengthening studies on the bioavailability and bioaccessibility of PUFA raw materials in the body; (4) intensifying studies on the dose–effect relationship of complementary effects of PUFA raw materials in alleviating visual fatigue. At the same time, more in-depth research should be conducted on the physiological causes of visual fatigue to better boost the application of PUFAs raw materials in alleviating visual fatigue. In addition, in future research, the ratio of ω-6/ω-3 in alleviating visual fatigue should also be given more attention and research.

## Figures and Tables

**Table 1:** Levels of ω-3 and ω-6 PUFAs in foods.

Category	Name	Total PUFAs	ω-3 PUFAs			ω-6 PUFAs		Ref.
			ALA (18:3)	EPA (20:5)	DHA (22:6)	LA (18:2)	AA (20:4)	
Nuts	Almond	9.93~26.66	0.07~0.29	/	/	8.17~26.66	/	[37,38]
	Walnut	72.66~77.48	12.61~16.92	/	/	58.64~63.74	/	[37,39]
	Hazelnut	7.30~11.48	0.08~0.12	/	/	7.20~11.25	/	[37,40]
Fish	Blowfish	30.20~41.80	0.10~0.40	1.30~6.90	14.00~26.70	1.40~10.60	7.00~12.20	[41]
	salmonids	49.18~49.28	0.62~1.62	3.86~6.37	27.65~28.88	9.41~11.81	2.12~2.30	[42]
	Cod	59.00~62.00	0.18~0.32	0.07~0.09	30.00~31.00	0.50~0.70	2.40~6.00	[43]
Seed	Chia seed	24.77~28.37	17.8~19.55	/	/	5.25~5.84	/	[44,45]
	Flaxseed	70.29~71.65	55.72~57.93	/	0.11~0.14	13.58~14.68	/	[46]
	Sunflower	58.90~61.80	0.06~0.11	/	/	58.83~61.68	<0.01	[46]
Seaweed	Red algae	/	0.20~0.50	27.80~45.40	/	0.60~1.60	5.30~23.40	[47]
	Brown algae	/	2.10~9.70	3.10~13.20	/	3.70~9.90	7.90~18.60	[47]
	Green algae	/	0.50~21.90	1.00~1.40	/	2.30~28.60	0.30~1.20	[47]
Meat	Beef	9.48~12.52	0.13~0.25	2.87~3.71	/	0.80~1.84	0.06~0.24	[48]
	Lamb	11.01~1.60	1.25~1.35	0.60~0.70	0.59~0.69	/	1.87~2.19	[49]
Oil	Argan oil	1.90~3.90	0.18~0.30	/	/	1.60~3.20	/	[50]
	Fish oil	/	1.04~1.37	6.21~8.13	12.80~13.30	1.32~1.67	0.85~0.95	[51]
	Soybean oil	/	5.50~6.62	/	/	50.9~57.10	/	[44,52]
	Flaxseed oil	67.40~68.27	53.40~54.33	/	/	13.94~14.20	/	[44,53]

/ = Not mentioned; ALA = alpha-linolenic acid; EPA = eicosapentaenoic acid; DHA = docosahexaenoic acid; LA = linoleic acid; AA = arachidonic acid.

**Table 2:** The adequate intake (AI) of ω-3 PUFAs and ω-6 PUFAs by national authorities.

Country/Institution	Adult Male		Adult Women		Ref.
	ω-3, g/d (ALA)	ω-6, g/d (LA)	ω-3, g/d (ALA)	ω-6, g/d (LA)	
United States (NIH)	1.6	14–17	1.1	11–12	[79]
Europe (EFSA)	0.5% E	4.0% E	0.5% E	4.0% E	[80]
Australia and New Zealand (NHMRC)	1.6	14–17	1.1–1.3	11–13	[81]
China (NHC)	0.6% E	4.0% E	0.6% E	4.0% E	[82]
Japan (MHLW)	(2.0–2.4) ^a^	(8–11) ^a^	(1.1–1.3) ^a^	(7–9) ^a^	[83]
Canada (Health Canada)	1.3	13	0.8	8	[84]

E = Energy percentage. ^a^ Total for the whole day. NIH = National Institutes of Health; EFSA = European Food Safety Authority; NHMRC = National Health and Medical Research Council; NHC = National Health Commission of the People’s Republic of China; MHLW = Ministry of Health, Labour, and Welfare; ALA = alpha-linolenic acid; LA = linoleic acid.

**Table 3:** Mechanisms by which PUFAs reduce the extent of damage to the ocular surface and fundus, exerting their effects of the relief of visual fatigue.

Subjects/Animals	Subjects	Periodicity	Results	Conclusions/Potential Mechanisms	Ref.
People with visual fatigue	EPA: 162 mg/d/ DHA: 783 mg/d and lingonberry extract and lutein	4 w	Omega-3-rich dietary supplements can safely improve the subjective symptoms of eye strain and mental fatigue in humans.	Dietary supplements rich in omega-3s can help alleviate the onset of visual fatigue.	[28]
Dry eye mice	ALA or LA	5~10 d	Increased corneal fluorescein staining; increased corneal IL-1 β and TNF-α expression; and conjunctival IL-1 β, TNF-β, IFN-β, IL-2, IL-6 and IL-10. The ALA group, on the other hand, showed significantly reduced corneal fluorescein staining, corneal IL-1 and TNF-α expression and a significant reduction in conjunctival TNF-α associated.	ALA helps to relieve dry eyes and eye discomfort, and it reduces the expression of inflammatory factors on the ocular surface, thus relieving eye strain symptoms such as dry eyes.	[91]
Dry eye people	EPA: 1680 mg/d/ DHA: 560 mg/d	2 m	Two months of omega-3 supplementation significantly improved the subjective symptom scores of OSDI and DEQ in the dry eye population, significantly inhibiting the increase in levels of the ocular surface pro-inflammatory factor MMP-9.	Omega-3 may have alleviated the onset of dry eye by reducing ocular surface inflammation.	[92]
Dry eye people	EPA: 360 mg/d/ DHA: 240 mg/d	1 m	One month after the ω-3 intervention, the dry eye population showed significant improvement in TBUT, OSDI and Schirmer scores than the placebo group. The intervention and placebo groups showed significant differences in TBUT with changes of 71%, and 3.3%, dry eye symptoms changes of 26% and 4% and Schirmer score changes of 22.3% and 5.1%, respectively.	Omega-3 is effective at reducing the rate of tear evaporation; improving dry eyes and eye discomfort and effectively increasing tear production.	[93]
Dry eye people	EPA: 1680 mg/d/ DHA: 560 mg/d	12 w	At week 6, the ω-3 intervention group showed a significant reduction in tear osmolarity. At 12 weeks, both ω-3 index levels and TBUT were significant at 12 weeks, both ω-3 index levels and TBUT significantly improved and had reduced ocular surface MMP-9 expression.	ω-3 was associated with statistically significant improvements in tear osmolality, ω-3 index levels, TBUT, MMP-9 and OSDI symptom scores.	[95]
Rats with high eye pressure	Omega-3 PUFAs group: EPA + DHA ω-6 PUFAs group: GLA ω-3 + ω-6 PUFAs group: EPA + DHA + GLA	6 m	Significant activation of glial cells was observed in the eyes of control rats with high IOP, while animals in the ω-3 + ω-6 PUFAs group diet showed significant improvement.	The results of the study showed that the effects of the combination of ω-3 + ω-6 PUFAs were more effective than single supplementation in preventing retinal cell structure and glial cell activation induced by elevated IOP in rats.	[96]
Dry eye people	EPA: 427.5 mg/d/ DHA: 285 mg/d and other vitamins and zinc	3 m	ω-3 significantly reduced the percentage of HLA-DR-positive cells.	Supplementation with omega-3 may reduce the expression of HLA-DR conjunctival inflammatory markers and may help improve dry eye and ocular discomfort.	[97]
Rabbit with retinal damage	Algae oil (contains 33.34% DHA)	3 w	The intervention group significantly reduced the levels of IL-1β, TNF-α, IL-8 and COX-2 in the retina, downregulated NF-κB expression and significantly attenuated light-induced retinal apoptosis and neovascularization.	Diets containing omega-3 PUFA may protect against visible light-induced retinal damage. The mechanism may be related to reducing the expression of inflammatory factors in the damaged retina.	[105]
People with visual fatigue	Flaxseed oil capsules	30 d	After 30 d, the test food group showed a significant reduction in visual fatigue and a 70.3% improvement in the persistence of binocular vision, with a mean improvement of 11.2 ± 7.3% and an overall effective rate of 51%.	It is shown that omega-3 rich flaxseed oil softgels are effective in relieving human visual fatigue.	[106]
AMD crowd	EPA- and DHA-rich diet	3 m	Higher intakes of EPA and DHA prevented or delayed the onset of moderate AMD, but there appeared to be only a trend towards improvement for late-stage ADM. The results of the study were not available but not with significant differences.	Higher intake of EPA and DHA may prevent or delay the onset of moderate AMD	[113]

w = Week; d = day; m = month; ALA = alpha-linolenic acid; LA = linoleic Acid; TNF-α = tumor necrosis factor-α; IL-1β = interleukin-1β; TNF-β = tumor necrosis factor-β; IFN-β = interferon-β; IL-2 = interleukin-2; IL-6 = interleukin-6; IL-10-interleukin-10; DHA = docosahexaenoic acid; EPA = eicosapentaenoic acid; OSDI = ocular surface disease index; DEQ = dry eye questionnaire; MMP-9 = matrix metalloproteinase-9; Nrf2 = nuclear factor erythroid 2-related factor 2; Heme HO-1 = Oxygenase-1; COX-2 = cyclooxygenase-2; NF-κB = nuclear factor kappa-B; AMD = age-related macular degeneration.

## Data Availability

Not applicable.
